# Climate change and maritime security narrative: the case of the international maritime organisation

**DOI:** 10.1007/s13412-018-0509-2

**Published:** 2018-08-02

**Authors:** Basil Germond, Fong Wa Ha

**Affiliations:** Department of Politics, Philosophy and Religion, Lancaster University, Lancaster, LA14YL UK

**Keywords:** Global warming, Maritime criminality, Migration, IMO, Discourse, Corpus linguistics

## Abstract

Both climate change and maritime security are currently ranking high on states’ and international organisations’ political and governance agendas. However, academics and practitioners alike have hardly tackled the actual interlinkages and dependencies between the two issues. Taking the International Maritime Organization (IMO) as a case study, this article pioneers the use of corpus linguistic method to unravel the nonexistence of a narrative linking climate change impacts and the occurrence of maritime criminality despite some connections in practice. However, direct narrative links between climate change and migration as well as migration and maritime security were found, which can point at an indirect link between climate change and maritime security. The article concludes on the implications of these findings for academics and practitioners alike. The latter are encouraged to reflect on their current narrative in a bid to contribute to a better acknowledgement of the existing links between the impacts of climate change on natural and human systems and aspects of maritime security.

## Introduction

Since the turn of the twenty-first century, political actors such as states and international organisations have developed and promoted a discourse that links climate change and security (e.g. Commission of the European Communities 2009; UN Security Council 2007; Department of Defense 2015). The extent, significance and practical implications of this discourse have generated many academic studies (e.g. Parsons 2010; Scott 2008, 2012; Trombetta 2008; Von Lucke et al. 2014; on the actual links between climate change and security, see notably Barnett 2003; Barnett and Adger 2007; Gemenne et al. 2014; Gleditsch 2012; Hsiang and Burke 2014; Scheffran et al. 2012). At the same time, the expansion of the security agenda and 9/11 have generated fears about the power of nuisance of non-state actors operating at sea, such as terrorists, pirates, smugglers and even illegal fishers (e.g. African Union 2012; Council of the European Union 2014; French Government 2015; HM Government 2014). The upsurge of piracy at the Horn of Africa in 2007/08 has also strengthened this trend. This has resulted in a growing narrative emphasising the need to control ocean space (Germond 2015), which translates into maritime security practices by states and international organisations, such as counter-piracy and counter-human smuggling operations, maritime surveillance and the adoption and implementation of dedicated norms and regulations.

Despite the existence of a narrative on climate change and security and a narrative on maritime security at the highest level of decision-making, there is currently no known/visible narrative linking climate change impacts and the occurrence of maritime criminality. Academics and practitioners alike seem to have somewhat neglected the actual interlinkages between the two sets of issues. In other words, the links and dependencies between climate change and maritime security have not been the focus of many studies (rare examples include Cordner 2010; Jasparro 2009; Jasparro and Taylor 2008; Kaye 2012; Mazaris and Germond 2018; Rahman 2012; Rahman and Tsamenyi 2010). Against this backdrop, this article aims to unravel the extent to which this narrative is indeed absent from political discourses or whether there are embryonic signs of its development. To clarify this matter, we carry out a hybrid corpus and discourse analysis of the International Maritime Organisation (IMO) public documents/website (c.f. methodology section for a justification of the case study).

The main research question consists in searching the extent to which a narrative linking climate change and maritime security/criminality can be found in the IMO textual production. Then, if this narrative exists, how is the relationship conceptualised/presented? Can we find some ‘vectors’ linking climate change and maritime criminality, such as coastal populations’ vulnerability or sustainable development? If it does not exist, then how to explain the absence of such a discourse? How does the conceptualisation of climate change on the one hand and of maritime security on the other hand contribute to this lack of interlinkages in narrative? The findings will contribute to the academic effort consisting in fostering the recognition of complex interlinkages between climate change and maritime security, while helping practitioners reflecting on their priorities and on the extent to which they frame their responses to the two issues in a separate way.

## Methodology

A corpus linguistics approach has been considered as the most relevant approach for the research question, since it allows demonstrating narrative trends beyond the existence of disparate sentences and in a systematic way, reducing “the rich chaos of language [to its] boiled down extract” (Scott and Tribble 2006: 6). Quantitative data such as frequency lists and statistics can be extracted from the dataset, which allows demonstrating the existence or absence of narrative patterns and highlighting their particular linguistics characteristics. Billions of words can be processed meaning that the entire textual production of a given actor can be analysed, revealing patterns that would hardly be discernible otherwise.

The corpus of this study is sourced from the published IMO documents (freely available on the organisation’s public website). We chose this organisation because the IMO is the leading international institution which deals with maritime affairs. It has interests and competencies in both climate change at sea and in maritime security issues. The IMO describes itself as “the global standard-setting authority for the safety, security and environmental performance of international shipping. [Its] main role is to create a regulatory framework for the shipping industry that is fair and effective, universally adopted and universally implemented” (IMO website-About). In other words, the IMO is a regulatory and policy setting institution. Both “sustainable maritime development” and “maritime security” are ranked high on its agenda (ibid). Therefore, the IMO website is a suitable and representative source for a small-scale pioneer research project like the present study, which explores the linkages between climate change and maritime security.

To investigate these linkages, we looked at the collocations of the node words, i.e. the search words, related to these two issues. The concept of collocation in corpus linguistics has been researched for at least 60 years. It was first introduced as a technical term by Firth (1957), who later defined collocation as “statements of the habitual or customary places” (Firth 1968: 181) of a given word. To date, Firth’s view that collocation is a frequent co-occurrence of patterns of two lexical items has been widely accepted by corpus linguists (e.g. Sinclair 1991; Hoey 1991; Stubbs 1995; Hunston 2002; McEnery et al. 2006).

As this study aims to look at how strongly climate change is linked to maritime security in the IMO textual production (c.f. below for a discussion of the building of the corpus), it is fundamental to examine how frequent node words of one group co-occur with node words of another group (e.g. *climate change* vs. *maritime security*) in the corpus collected. In other words, the connections between individual collocates should be determined.

We have manually created a dataset with all documents and webpages containing references to both climate change (or global warming) and maritime security (or maritime criminality, piracy, illegal fishing, etc.). As the discussions which connect security and the maritime domain beyond naval developments only started at the turn of the new millennium (Bueger 2014; Germond 2015), we have only included documents and webpages dated after year 2000. Some documents, which were irrelevant to the study and created ‘noise’, have been manually removed from the corpus: technical guidelines, promotional documents, organisational procedures, policies, and action plans, lists of internet links to sources of information and Powerpoint presentation slides. The final corpus contains 1419 documents and webpages, with a total of 3,705,927 tokens obtained. For the purpose of this analysis, “token” is roughly equivalent to “word”.

After the corpus has been constructed, we carried out a manual qualitative analysis on a sample of randomly selected files from the corpus, so as to identify and select a list of relevant node words relating to *climate change* or *maritime security*. We also found a few common words of non-criminal maritime issues, i.e. pollution, accident and environmental protection, to serve as the control group. These node words are listed in Table 1.

**Table 1:** Selected node words relating to climate change or maritime security

‘Climate change’ group	‘Maritime security’ group	Control group
Climate change*	Maritime security*	Pollution*
Climatic change	Maritime cyber security	Maritime accident*
Global warming	Maritime cyber risk*	Marine environmental protection*
	Maritime criminality	
	Unlawful acts*	
	Piracy*	
	Armed robbery*	
	Human trafficking*	
	Smuggling*	
	Illegal fishing	
	Counter-terrorism*	

Node words with an asterisk are the restricted ones

In the starting phase, we formed all possible word pairs by pairing a node from the climate change group and a node from the maritime security group. For example, *climate change* and *maritime security*, *climate change* and *unlawful acts*, *global warming* and *piracy*, etc. As a control measure, we also paired up nodes from the climate change group with nodes from the non-criminal group. Collocations of these control pairs were examined as well.

To proceed with corpus analysis, we used LancsBox, which is a newly developed software and one of the very few corpus linguistics tools which supports the investigation of collocation in context, i.e. collocation networks. It visualises the collocations in the form of graphs of the network of words that collocate with each node word; by this means, the relationship between a node word and its textual environment can be revealed (Brezina et al. in prep; Brezina et al. 2015). Few other corpus linguistics tools, for example Wordsmith Tools (http://www.lexically.net/wordsmith/), also enable the building of collocation networks. However, the process involves mainly manual comparison of the associations between the keyword and its collocates (ibid). LancsBox, on the other hand, builds the networks automatically and therefore is ideal for the present study, which investigates predominantly the collocation networks formed by the two groups of node words relating to *climate change* and *maritime security*. It enables us to have an insight into lexical interconnections between the two topics.

Based on the word pairs of Table 1, we first identified collocates to each node, which are the words that co-occur with the node. We then used the mutual information (MI) statistic to choose the most pertinent words nodes by selecting a cut-off frequency of 5, a cut-off statistical value of 6 and a span of ± 5 words regardless of punctuation. The cut-off frequency is the number of times the token appears in the corpus for it to be included in the graph. For this study, any token with a frequency below 5 is considered too rare to be taken into consideration. The collocates must be within a span of five words to the left or right of the keyword. The associate measure MI score is a common measurement tool used in corpus linguistics. It is used for this research because it favours strongly related low-frequency collocates (e.g. displacement, risk, etc.) over highly frequent but loosely associated collocates (e.g. the, in, of, etc.). Application of the method described above led to remove from the search nodes of Table 1 those with none or very few collocates. The final node words retained are asterisked in Table 1.

**Fig. 1:**
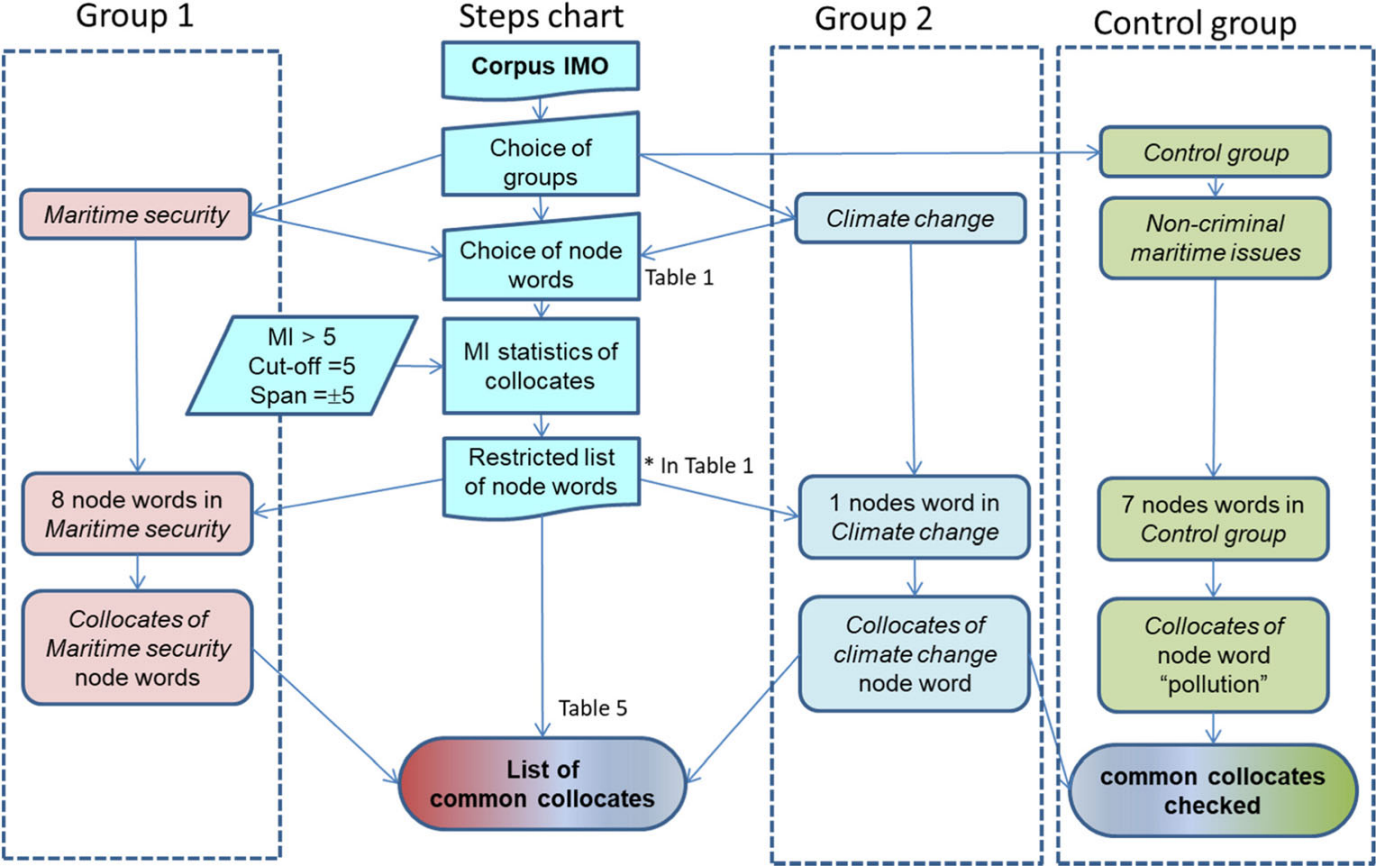
Diagram of the corpus linguistics methodology used (The column designed as Group 1, resp. Group 2, resp. Control group represents the Maritime security group, resp. the Climate change  group, resp. the Non-criminal maritime issues control group. The column ‘Steps chart’ displays the successive steps of the method to obtain a list of common collocates.)

Finally, using LancsBox GraphColl function, we investigated whether the narrative demonstrates a causality link (or at least a link) between the impacts of climate change at/or from the sea and the occurrence of maritime criminality/maritime security issues. We started by independently analysing the main collocates of *climate change* and *maritime security*, so as to get a picture of both narratives separately. We then tried to find out how *climate change* and *maritime security* were interrelated including through an indirect link between climate change and maritime security such as via the discussions of (illegal) migration. The complete methodology is summarised by the organigram of Fig. 1.

### Data and analysis

We first used the GraphColl function of LancsBox to search for the collocates of each node word listed in Table 2. GraphColl visualises how strong the collocation is, how frequent the collocates appear, as well as the position of the collocates. This was performed in three steps:

**Table 2:** Statistics of the collocates of *climate change*

Position	Collocate	MI score	Frequency (coll.)	Frequency (corpus)
R	warming	12.37	17	30
R	adaptation	11.96	18	42
R	cop	11.79	11	29
R	unfccc	11.64	12	35
R	mitigation	11.46	26	86
R	degradation	11.41	12	41
L	paris	10.50	22	141
L	mitigate	10.40	15	103
L	atmospheric	10.24	10	77
R	displacement	9.62	6	71
L	debate	9.34	15	215
L	combat	9.30	11	163
L	disaster	9.04	7	124
R	impacts	8.99	17	311
R	goal	8.44	9	242
L	urgent	8.32	6	175
L	responding	8.17	5	162
L	gender	8.06	6	210
L	framework	8.02	36	1294

Only collocates with MI > 8 are shown. Underlying data source: IMO public website

#### Step 1: Collocates of climate change

**Fig. 2:**
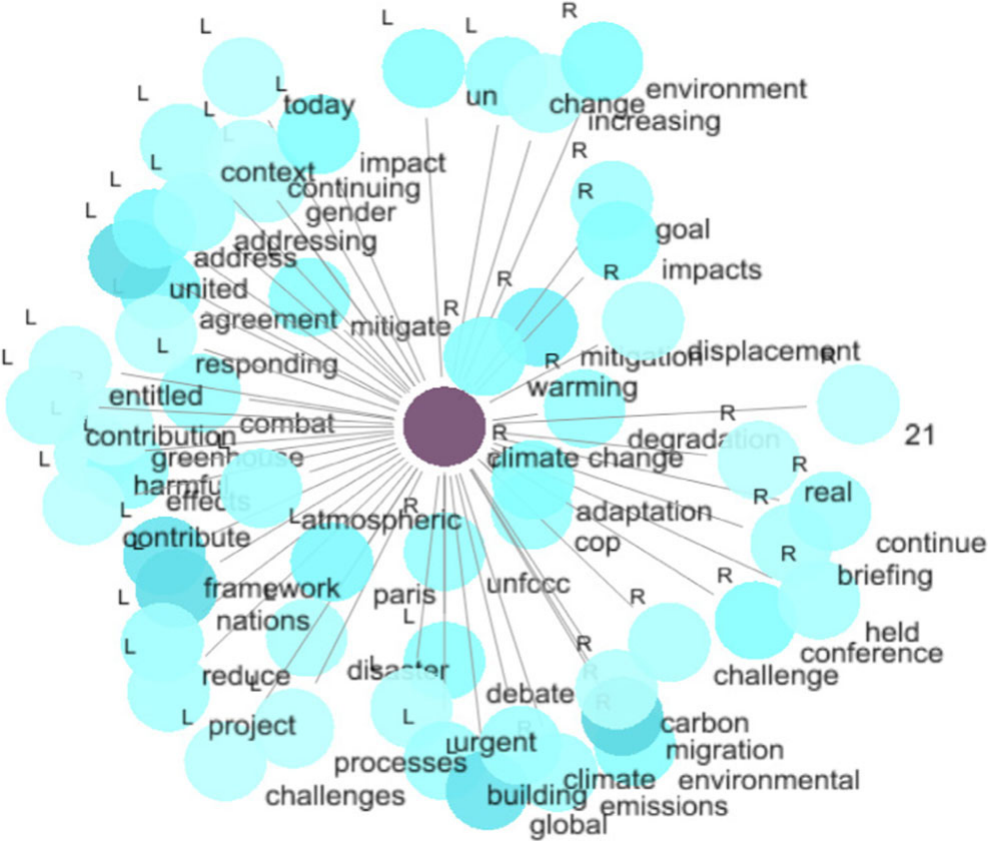
Graph for the collocates of *climate change* (All network graphs presented in this article correspond to computer screenshots as they are displayed by the LancsBox software. Underlying data are from the IMO public website.)

Figure 2 shows the visualisation of the collocates of *climate change*. Collocates which are closer to the node word are stronger than the ones that are further apart. Collocates with a darker colour in the dot are more frequent than the ones with a lighter colour. The positions (R or L) of the collocates on the graph echo their positions on the concordance lines as well. Concordancing is “a means of accessing a corpus of text to show how any given word or phrase in the text is used in the immediate context in which it appears” (Flowerdew 1996, p.87). A concordance line is a line from a text of the corpus which contains the node word to be studied. Data show that climate change in the IMO narrative is mainly associated with (1) institutional processes/frameworks (e.g. <cop>, <conference>, <unfccc>, <framework>, <united nations>) and (2) policy requirements/settings (e.g. <adaptation>, <mitigation>, <address/addressing>, <responding>, <reduce>, <combat>, <urgent>). This fits with the technical discourse adopted by international organisations in the field of environment and development (e.g. Ferguson 1990) as well as a tendency to favour short-term, problem-solving approaches.

Four interesting collocates appear when it comes to negative impacts of climate change: <disaster>, <gender>, <displacement> and <migration>. Disaster may refer to the effects of climate change on natural ecosystems but also on human systems, since an increased frequency of natural disasters (especially in the poorest regions of the world) can negatively impact on human security via health and food supply issues. Also, extreme weather events and disasters can damage the maritime economy and negatively impact on food security (Allison et al. 2009). The reference to gender issues may well point at the supposed gender dimension of the impacts of climate change, i.e. women are either more vulnerable to these effects or, on the contrary, better prepared to respond to these changes (for a critical discussion of these propositions, see Arora-Jonsson 2011). Displacement and migration are interesting collocates, since, represented as potential negative outcomes of climate change, they can point towards an indirect link between climate change and maritime security narratives, which will be further discussed below.

Table 2 lists statistics of the collocates of *climate change*. The ‘Position’ column shows whether the collocate is located on the left or right of the node word *climate change* on average. ‘Frequency (corpus)’ shows the total frequency the collocate appears in the corpus. ‘Frequency (coll.)’ shows the frequency of its collocation with the node word *climate change*. It is worth noting that the collocates are arranged in descending order of significance, given by the MI scores. We can see that the 10 most strongly collocated words are indeed a mixture of institutional processes (i.e. <cop>, <unfccc>), policy requirements (i.e. <adaptation>, <mitigation>, <mitigate>) and negative impacts of climate change (i.e. <warming>, <degradation>, <displacement>). This mirrors findings from Fig. 2.

#### Step 2: Collocates of maritime security

**Fig. 3:**
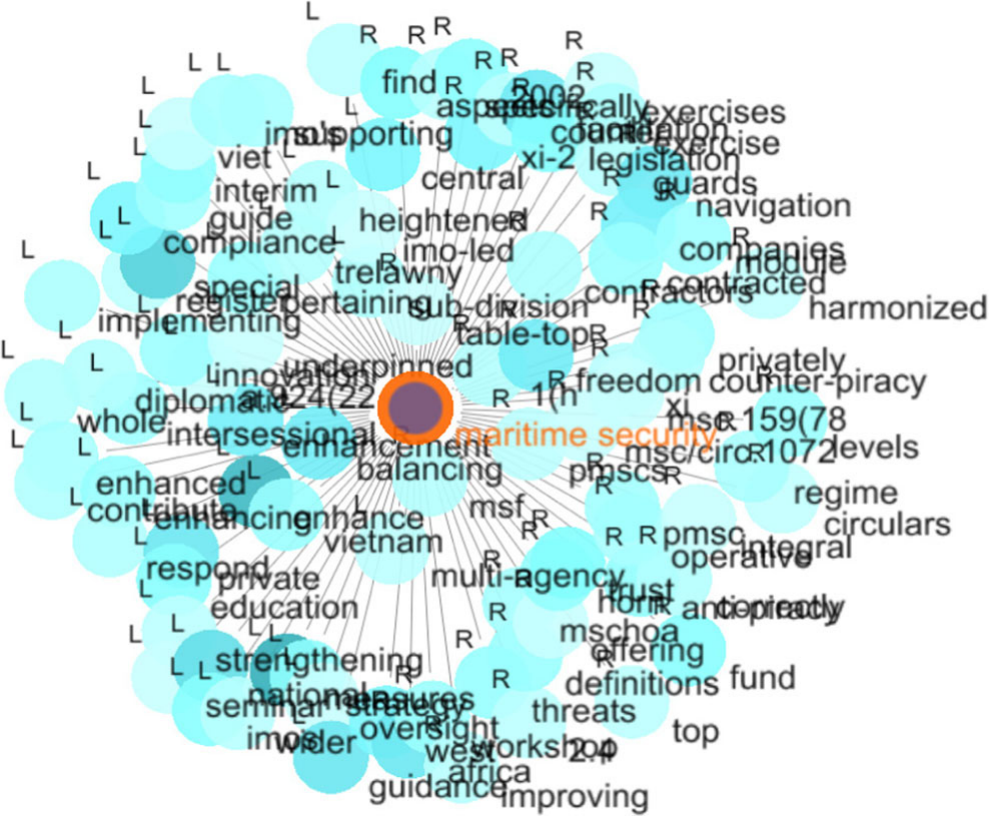
Graph for the collocates of *maritime security*

The second step consisted in analysing the collocates of the node word *maritime security* (c.f. Fig. 3). It appears that maritime security in the IMO narrative is mainly associated with (1) the need to develop or improve relevant institutional frameworks (e.g. <contractors/contracted>, <diplomatic>, <fund>, <guards>, <imo-led>, <multi-agency>, <mschoa> (Maritime Security Center Horn of Africa), <private/privately>, <regime>, <workshop>, (2) policy settings (e.g. <compliance>, <definition>, <education>, <guidance>, <innovation>, <legislation>, <register>), and (3) generic calls for action and cooperation/coordination (e.g. <enhanced/enhancing> <guide/guidance> <harmonized>, <implementing>, <improving>, <respond>, <strengthening>, <supporting>). The only collocate to another node word from the *maritime security* group is <counter-piracy>. Statistics of collocates of *maritime security* can be seen in Table 3.

**Table 3:** Statistics of the collocates of *maritime security*

Position	Collocate	MI score	Freq (coll.)	Freq (corpus)
R	msf	11.44	9	9
R	1(h	11.29	9	10
R	table-top	10.96	5	7
L	balancing	10.95	57	80
R	pmscs	10.66	7	12
R	freedom	10.43	57	115
L	sub-division	10.32	11	24
L	multi-agency	10.16	7	17
R	msc/circ.1072	9.76	5	16
L	vietnam	9.76	19	61
L	enhancement	9.75	54	174
L	enhance	9.56	226	835
L	underpinned	9.52	5	19
R	contractors	9.32	11	48
L	imo-led	9.30	5	22
L	trelawny	9.15	9	44
R	xi	9.02	8	43
R	msc.159(78	9.01	5	27
L	a.924(22	8.96	5	28
L	pertaining	8.91	19	110
R	trust	8.84	35	212
R	horn	8.80	22	137
R	mschoa	8.73	14	92
R	pmsc	8.68	15	102
L	heightened	8.52	9	68
L	innovation	8.41	17	139
R	operative	8.34	12	103
R	counter-piracy	8.18	10	96
R	offering	8.15	5	49
R	contracted	8.12	19	190
L	central	8.06	42	438

Only collocates with MI > 8 are shown. Underlying data source: IMO public website

**Fig. 4:**
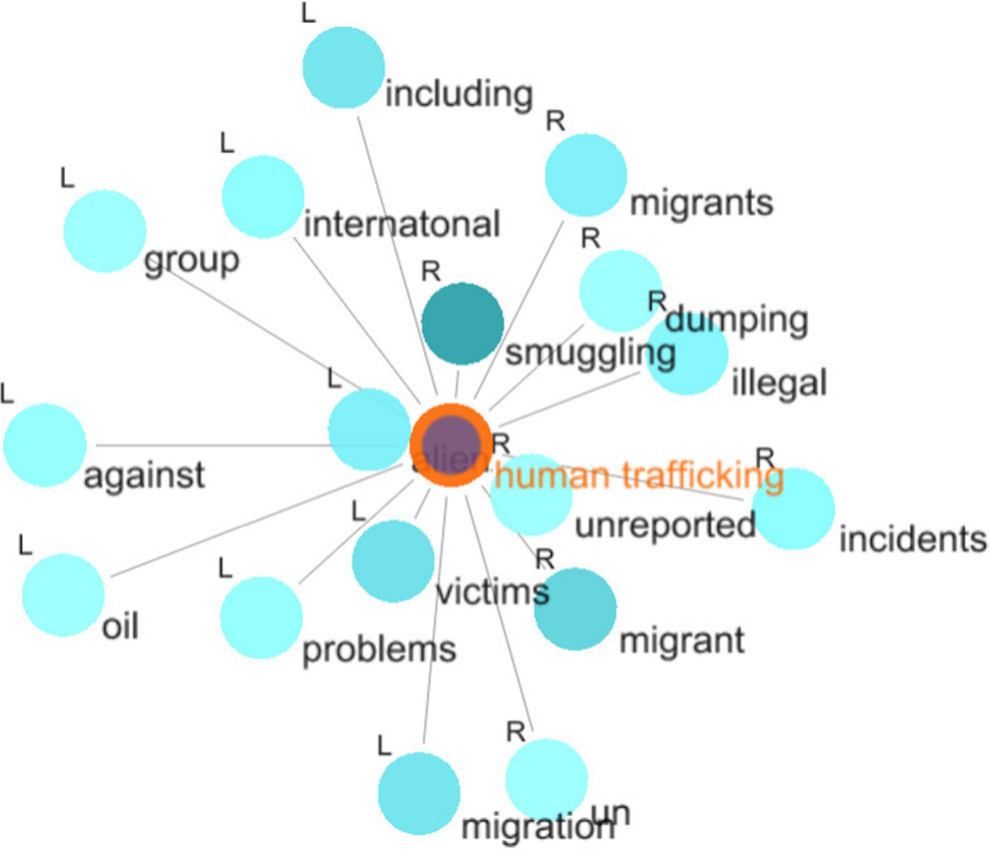
Graph for the collocates of *human trafficking*

The investigation of collocates of the node word *human trafficking* (also from the *maritime security* group) shows that <migrant/s> and <migration> are the most frequent collocates (besides <smuggling>) (c.f. Fig. 4 and Table 4). Unsurprisingly, <migrant> is among the most collocated words to *human trafficking*. This points to the fact that migration is both related to climate change and to maritime security in the IMO narrative, which will be further discussed below.

**Table 4:** statistics of the collocates of *human trafficking*

Position	Collocate	MI score	Freq (coll.)	Freq (corpus)
L	alien	13.16723	9	49
R	unreported	12.93395	5	32
*R*	*smuggling*	*12.34633*	*34*	*327*
L	victims	12.18575	12	129
*R*	*migrant*	*10.57933*	*15*	*491*
R	dumping	10.08845	5	230
R	illegal	9.597995	8	517
L	problems	9.50698	6	413
*R*	*migrants*	*8.554325*	*9*	*1199*
L	international	8.343891	7	1079
R	incidents	7.58569	7	1825
R	un	7.574194	5	1314
*L*	*migration*	*7.536172*	*11*	*2968*
L	including	6.638122	11	5531
L	against	6.357774	6	3664
L	group	6.335426	5	3101
L	Oil	6.151764	5	3522

Lines in italics refer to the most frequent collocates. Underlying data source: IMO public website

#### Step 3: Common collocates between climate change and each node word related to maritime security

**Fig. 5:**
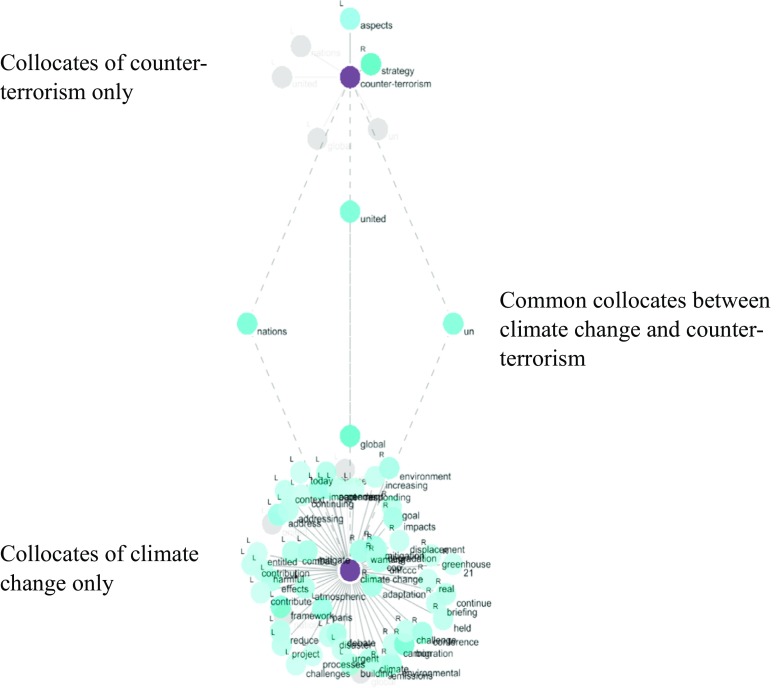
A graph of common collocates between *climate change* and *counter-terrorism*

The third step consisted in looking for direct narrative links between climate change and maritime security. This was done by looking at the common collocates between the node word *climate change* and each node word related to *maritime security* (words with an asterisk in Table 1). An example of visualising common collocates between two node words can be seen in Fig. 5 (counter-terrorism).

A list of common collocates was found regarding *climate change* and each node word relating to maritime security, i.e. *maritime security*, as well as *maritime cyber risk*, *unlawful acts*, *piracy*, *armed robbery*, *human trafficking*, *smuggling* and *counter-terrorism* (c.f. Table 5). However, there appeared to be very limited direct narrative links between climate change and maritime security.

**Table 5:** List of common collocates of *climate change* and node words of maritime security

Node word	Common collocate	Frequency (coll)
*Maritime security*	contribute	10
*Maritime cyber risk*	–	–
*Unlawful acts*	framework	7
*Piracy*	combat	39
*Armed robbery*	combat	12
*Human trafficking*	migration	11
*Smuggling*	combat	12
*Counter-terrorism*	united	11
	nations	11
	UN	10
	global	14

Underlying data source: IMO public website

As can be seen in Table 5, only eight common collocates between climate change and maritime security were found in the entire corpus of the IMO. Most pairs had only one common collocate. Logically, one of the node word pairs, *climate change* and *maritime cyber risk*, had no common collocates at all. The rare common collocates show a common emphasis on the institutional framework (<UN>, <united nations>, <framework>, <global>) and on the need to do something in a proactive way (<combat>). The most relevant common collocate (also found in Tables 3 and 4) was found in the *climate change* and *human trafficking* node word pair: <migration>. Indeed, as mentioned above, migration (and displacement) is also a collocate of climate change. Thus, the only occurrence of an indirect link between climate change and maritime security can be found via migration. The literature has suggested that climate change (negatively) impacts on food security and population well-being, then potentially generating (illegal) migration or human trafficking, with migrants travelling (or being trafficked) by sea (Jasparro and Taylor 2008; Kaye 2012), thus a link to maritime criminality and maritime security issues.

**Fig. 6:**
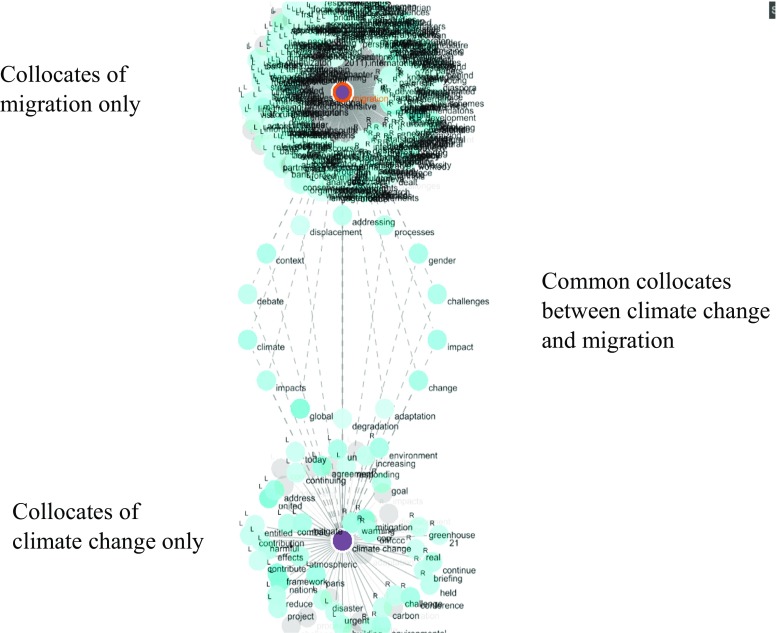
A graph of common collocates between *climate change* and *migration* (We needed to consider a large number of collocates before shared items appear. This speaks to the weakness of the narrative link between concepts but also hinders readability. However visual distinction between individual collocates is not required in statistical methodologies such as corpus linguistic. Such figures are visual representations of the complexity of lexical interconnections.)

**Fig. 7:**
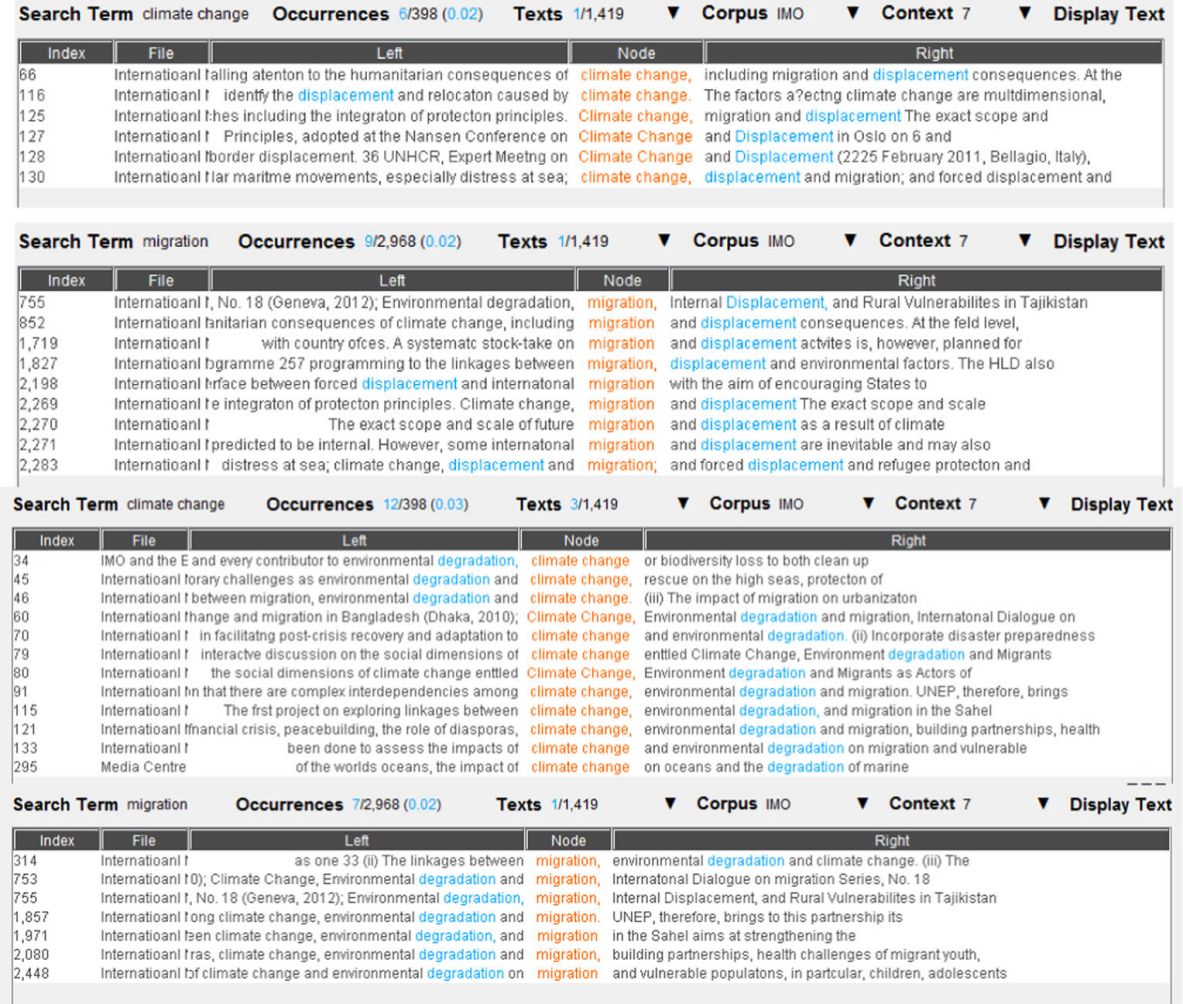
The concordance lines of <displacement> and <degradation> as a common collocate of climate change and migration. Underlying data source: IMO public website

Figure 6 shows a graph of common collocates between *climate change* and *migration*. In addition to the direct collocation between climate change and migration, common collocates do not indicate any more precise connection with maritime security. Building on this, Fig. 7 shows the concordances of some common collocates (i.e. <displacement>, <degradation>) between *climate change* and *migration* adding a qualitative insight. Examples of concordance tend to corroborate the existence of indirect links between climate change and maritime security via potential increased migration, although concordances also refer to land migration.

**Fig. 8:**
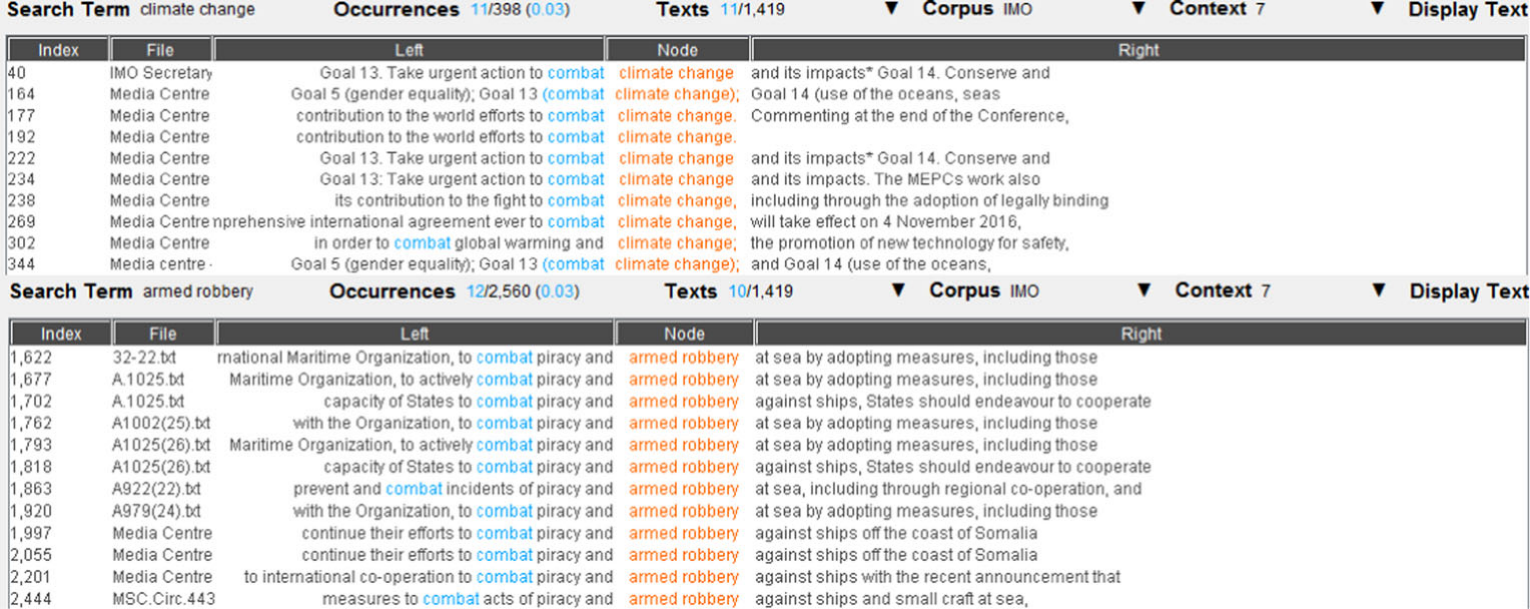
The concordance lines of *combat* as a common collocate of *climate change* and *armed robbery*. Underlying data source: IMO public website

**Fig. 9:**
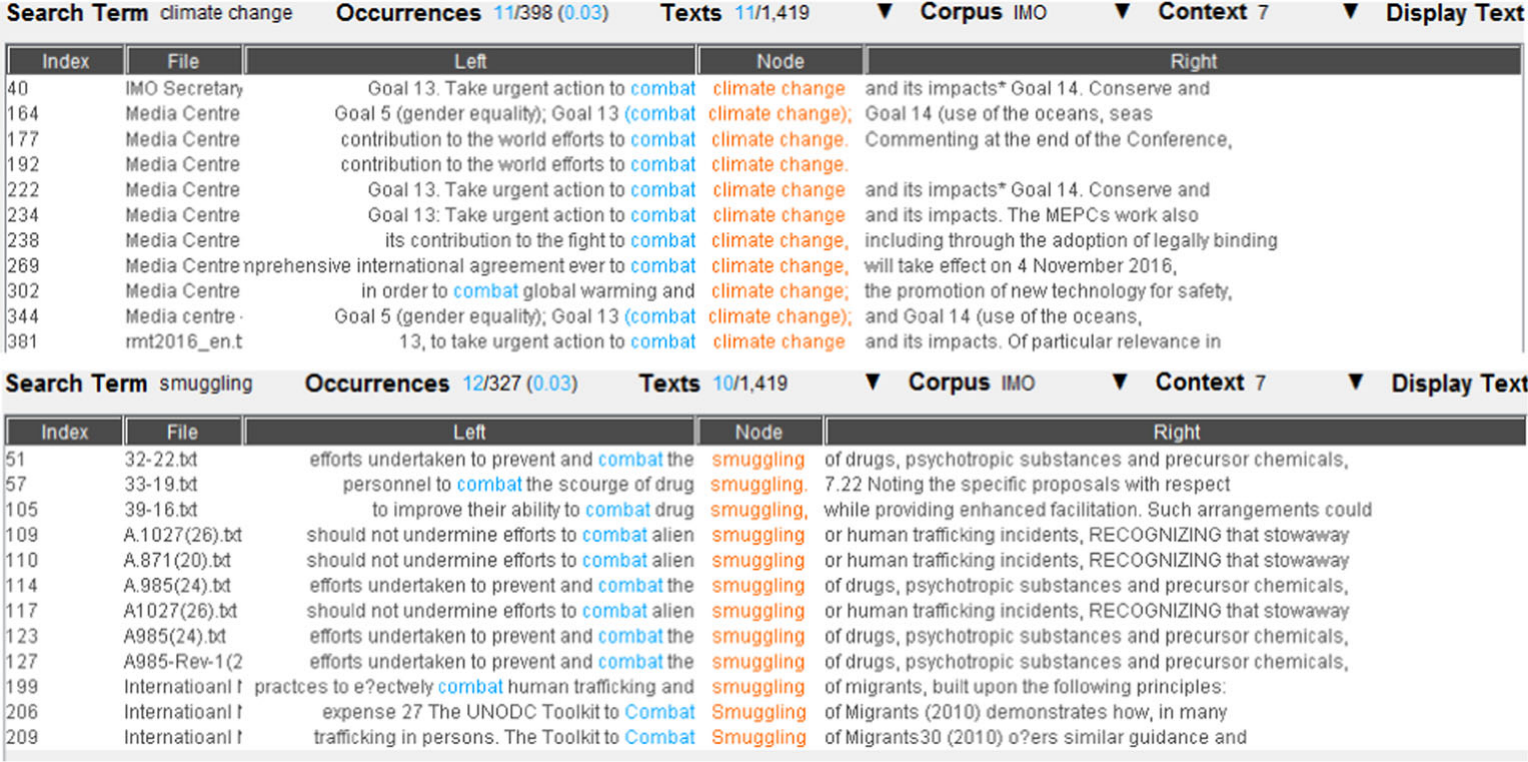
The concordance lines of combat as a common collocate of *climate change* and *smuggling*. Underlying data source: IMO public website

We then took a closer look at the concordance lines of each common collocate, in order to find further possible links between climate change and maritime security. Once again, the concordance lines of the eight common collocates did not show evidence of narrative links between climate change and maritime security. Figure 8 and Fig. 9, for example, present the concordance lines of *combat*, a common collocate of *climate change* and *armed robbery/smuggling*. None of the concordance lines show a link between *climate change* and *armed robbery/smuggling*, beyond the fact that the word <combat> is anyway used in two different lexical contexts, i.e. dealing with the threats posed by real criminals versus dealing with the negative impacts of climate change.

**Fig. 10:**
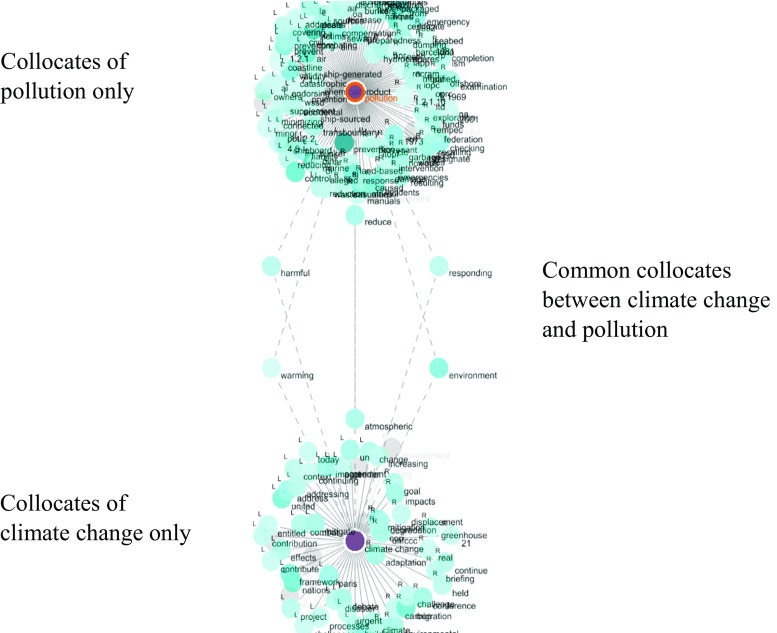
Common collocates between *climate change* and *pollution*

**Fig. 11:**
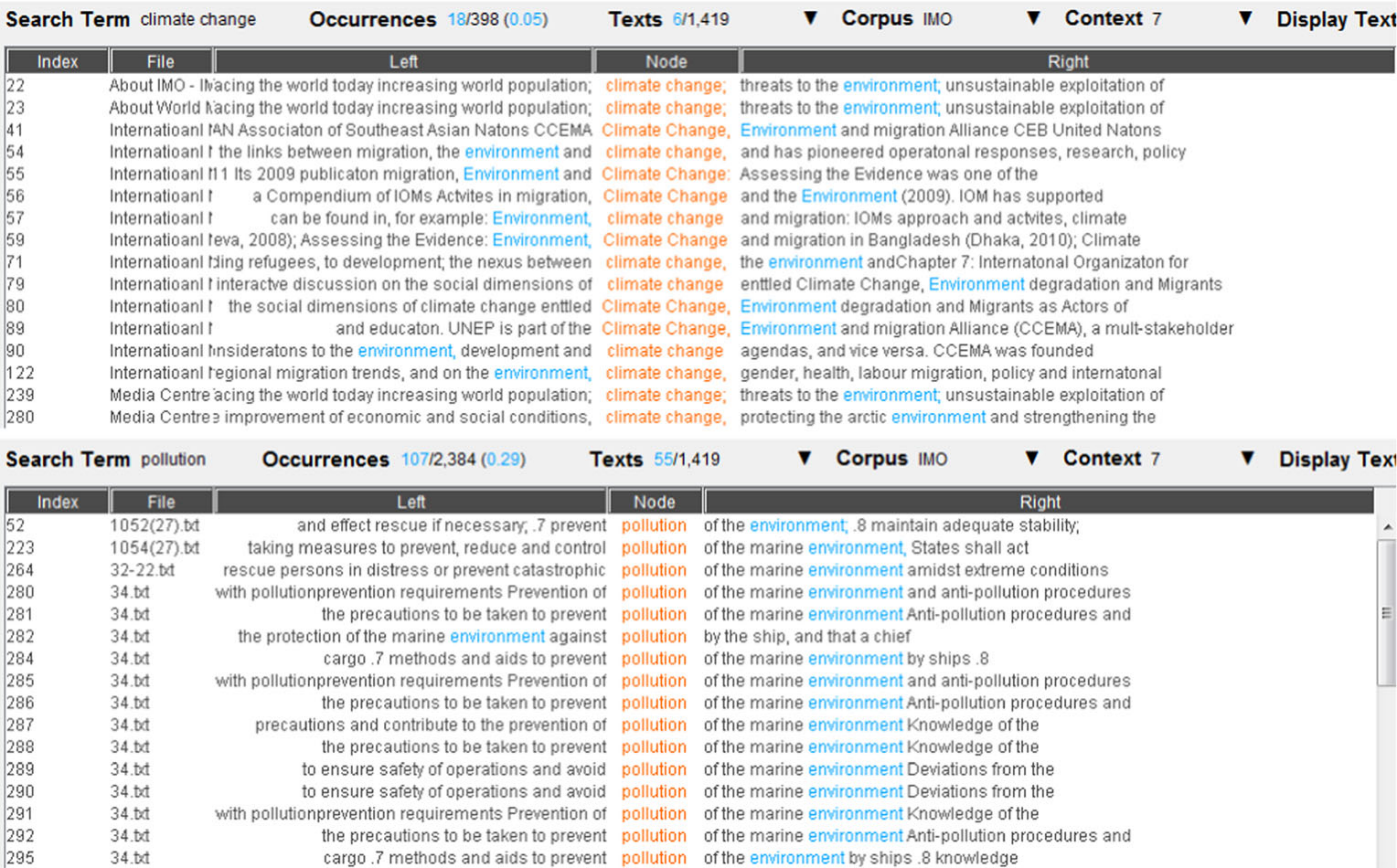
Concordance lines of *environment*, a common collocate of *climate change* and *pollution*. Underlying data source: IMO public website

On the other hand, however, links were observed between *climate change* and the node word *pollution* in the control group, which contains the node words related to non-security (i.e. safety) maritime issues. As we checked the concordance lines of the common collocates presented in Fig. 10 (i.e. <reduce>, <harmful>, <responding>, <warming>, <environment>, <atmospheric>), *climate change* and *pollution* were both linked to harmful environmental effects. For example, Fig. 11 shows the concordance lines of *environment*, a common collocate of *climate change* and *pollution*, in which climate change is directly addressed as a threat to the environment, in the same way pollution is. This confirms that there seems to be a stronger narrative link between climate change and non-security maritime issues, although connections between *climate change* and other node words in the control group (i.e. *marine environmental protection* and *maritime accident*) are not strong according to textual data.

## Discussion and conclusion

Using corpus linguistic methods, textual data show that, except for the indirect link between climate change and maritime security via migration/displacement, the IMO narrative does not encapsulate the interlinkages between climate change and maritime security. Despite having interests and responsibility in offering solutions to tackle both climate change and maritime security, the IMO does not seem to narratively represent the two issues (and the impacts of the two issues) as something linked or something that could potentially be linked, except indirectly in the case of climate change-induced migration. It is worth noting that findings might be limited by the following methodological constraint: the span of ± 5 (chosen in order to stay within typical linguistic structures and to reduce noise) does not take into account a potential narrative between climate change and maritime security that might be developed across entire paragraphs.

This reveals the need to start serious discussions with the help of both natural scientists and social/political scientists in a bid to initiate a reflexion on the existence of links and dependencies between the two issues. The existing literature has shown that the impacts of climate change on natural systems, such as a loss, or change in, marine biodiversity, can then reverberate on human, social and political systems, via economic slow-down, food insecurity, natural disasters, extreme weather events, forced displacements, vulnerability of coastal populations as well as the synergistic impacts of these processes. This can in turn increase the incentive to engage in maritime criminal activities (e.g. illegal fishing, piracy, human smuggling) as well as increase the risk of illegal immigration (e.g. Allison et al. 2009; Cinner et al. 2012; Cordner 2010; Jasparro 2009; Jasparro and Taylor 2008; Kaye 2012; Mazaris and Germond 2018; Perch-Nielsen et al. 2008; Pomeroy et al. 2016; Rahman 2012; Rahman and Tsamenyi 2010; Suárez de Vivero and Mateos 2017).

So long as actors tasked to tackle climate change concentrate on mitigation and adaptation (something that the IMO narrative seems to put forward), and so long as they do not include a reflexion on the impacts of damaged or threatened natural systems not only on food security, income and living conditions but also on the occurrence of (maritime) criminality, an important element of the puzzle will be missed. Our analysis has shown that this lack of interlinkages in narrative may be due to the very conceptualisation of climate change on the one hand and of maritime security on the other hand. In both cases, textual data shows that the vocabulary employed to conceptualise the two issues is very technical, ranging from institutional processes and frameworks, to policy requirements and settings, to generic calls for action and coordination. The specific framing of climate change within institutional processes and policy settings rather than a cause/consequence process involving issues at the societal level, whereas maritime security is conceptualised as a series of illegal practices that must be tackled, certainly explains the current lack of narrative linking the two issues. The origin of this technicality can be traced back to the IMO’s traditional role which is to improve the safety of the maritime shipping industry. In this organisational context, climate change and maritime security issues are likely to be associated to threats to maritime shipping, hence the problem-solving/technical approach of the IMO consisting in setting up rules, regulations, guidelines and agenda as well as promoting multilateral responses to transnational threats. This also seems to fit with the “technocratic and industry-oriented” nature of the IMO secretariat discussed by Campe (2009: 144).

Our findings have practical implications for both academics and practitioners. This article reveals the need for academics to find ways to conceptualise these dependencies between climate change and maritime security and to quantify the synergistic links between the two issues. It is crucial to work on better integrating indicators reflecting ecological risk (e.g. extreme weather events, loss of biodiversity, velocity of climate change), social vulnerability (e.g. capacity to adapt), exposure to impacts (e.g. localization on the global grid), economic consequences of climate change as well as maritime criminality indexes. Practitioners will benefit from such scientific advances, but they are also invited to move beyond an institutional processes/policy setting narrative so as to further account for the interlinkages between climate change and maritime security. Linking maritime security and climate change parallels the move from a problem-solving approach to one that deals with the underlying causes of maritime criminality, of which climate change is but one. This would help pushing forward the climate change-maritime security nexus agenda forward, which would eventually improve current ocean governance practices.
